# TGN-020 Alleviate Inflammation and Apoptosis After Cerebral Ischemia–Reperfusion Injury in Mice Through Glymphatic and ERK1/2 Signaling Pathway

**DOI:** 10.1007/s12035-023-03636-w

**Published:** 2023-09-11

**Authors:** Xiaohong Li, Zhuoxi Xie, Qian Zhou, Xiaoli Tan, Weiting Meng, Yeyu Pang, Lizhen Huang, Zhihao Ding, Yuanhong Hu, Ruhua Li, Guilan Huang, Hao Li

**Affiliations:** 1 Department of Neurology, Affiliated Hospital of Guilin Medical University, Guilin, 541001 China; 2 Department of Neurology, the Second Affiliated Hospital of Guilin Medical University, Guilin, 541199 China

**Keywords:** Ischemic stroke, Inflammation, RNA-seq, Glymphatic system, TGN-020

## Abstract

Post-stroke acute inhibition of aquaporin 4 (AQP4) is known to exacerbate inflammation and apoptosis, yet the underlying mechanisms are not fully understood. The objective of this study was to investigate the specific mechanism of inflammation and apoptosis following cerebral ischemia–reperfusion (I/R) injury using the AQP4-specific inhibitor, N-(1,3,4-thiadiazol-2-yl) pyridine-3-carboxamide dihydrochloride (TGN-020). Ischemic stroke was induced in mice using the middle cerebral artery occlusion (MCAO) model. The C57/BL6 mice were randomly divided into three groups as follows: sham operation, I/R 48 h, and TGN-020 + I/R 48 h treatment. All mice were subjected to a series of procedures. These procedures encompassed 2,3,5-triphenyltetrazolium chloride (TTC) staining, neurological scoring, fluorescence tracing, western blotting, immunofluorescence staining, and RNA sequencing (RNA-seq). The glymphatic function in the cortex surrounding cerebral infarction was determined using tracer, glial fibrillary acid protein (GFAP), AQP4 co-staining, and beta-amyloid precursor protein (APP) staining; differential genes were detected using RNA-seq. The influence of TGN-020 on the extracellular signal-regulated kinase 1/2 (ERK) 1/2 pathway was confirmed using the ERK1/2 pathway agonists Ro 67–7467. Additionally, we examined the expression of inflammation associated with microglia and astrocytes after TGN-020 and Ro 67–7467 treatment. Compared with I/R group, TGN-020 alleviated glymphatic dysfunction by inhibiting astrocyte proliferation and reducing tracer accumulation in the peri-infarct area. RNA-seq showed that the differentially expressed genes were mainly involved in the activation of astrocytes and microglia and in the ERK1/2 pathway. Western blot and immunofluorescence further verified the expression of associated inflammation. The inflammation and cell apoptosis induced by I/R are mitigated by TGN-020. This mitigation occurs through the improvement of glymphatic function and the inhibition of the ERK1/2 pathway.

**Supplementary Information:**

The online version contains supplementary material available at 10.1007/s12035-023-03636-w.

## Introduction

Ischemic stroke stands as one of the principal causes of death and disability in humans. Ischemia–reperfusion (I/R) may induce secondary neuropathologist, including inflammation and cerebral edema, potentially culminating in neuronal apoptosis in the ischemic core [1, 2]. As evidenced by numerous clinical trials and fundamental studies, the activation of microglia and astrocytes mediates the inflammatory response, which contributes to ischemic stroke injury [3, 4]. Furthermore, the build-up of neuroinflammation-associated amyloid-beta peptide (Aβ) around astrocytes in ischemic brain tissue not only results in brain I/R injury and dementia induced by ischemic stroke but also exacerbates cerebral I/R [5].

The glymphatic system is composed of periarterial cerebrospinal fluid (CSF) inflow that aligns with the direction of blood flow [6]. It facilitates the exchange of periarterial CSF with interstitial fluid (ISF) in the brain parenchyma through the perivascular space. The glymphatic system’s key characteristic is the intensive expression of aquaporin 4 (AQP4) in the astrocyte terminals, promoting CSF influx into the brain parenchyma and its mixing with ISF [7, 8]. Recent studies have demonstrated that the glymphatic system plays a pivotal role in maintaining brain homeostasis by assisting in the clearance of toxins, metabolic waste, and excess fluids from the brain parenchyma, including Aβ [9, 10]. Alteration in AQP4 polarization at the termini of perivascular astrocytes is identified as the primary cause of their dysfunction [11]. Dysfunction of the glymphatic system is closely linked to stroke. Studies have indicated that increased polarization of AQP4 can mitigate ferroptosis following subarachnoid cerebral hemorrhage [12]. Hyperplasia of inflammatory astrocytes significantly contributes to the alteration in AQP4 polarization. Studies have established that hyperplasia of inflammatory astrocytes plays a crucial role in the change of AQP4 polarization. Subsequent to cerebral I/R, alterations in AQP4 polarization and astrocyte proliferation lead to glymphatic dysfunction, resulting in the accumulation of metabolic waste products such as Aβ and inflammatory factors. This exacerbates brain pathological damage [7, 13–15]. Therefore, further research on the treatment of glymphatic system dysfunction after brain I/R injury is necessary. Extracellular signal-regulated kinase 1/2 (ERK) belongs to the mitogen-activated protein kinase family and plays a role in signaling cascades and delivers extracellular signals to intracellular targets [16]. Overactivation of proteins and kinases upstream in the ERK1/2 pathway has been implicated in various diseases, such as apoptosis, inflammation, developmental disorders, and neurological disorders [17–19]. AQP4 has the potential to affect inflammation and the ERK1/2 pathway; for instance, N-(1,3, 4-thiadiazol-2-acyl) pyridine-3-carboxyamide dihydrochloride (TGN-020) treatment can inhibit ERK1/2 phosphorylation by suppressing AQP4 expression [20]. TGN-020 treatment reduces astrocyte activation and the secretion of inflammatory cytokines tumor necrosis factor-a (TNF-α) and interleukin-1β (IL-1β) induced by spinal cord injury in rats by negatively regulating AQP4 [21]. However, the impact of AQP4 on the ERK1/2 pathway in brain I/R models has not been delineated in previous studies. Therefore, our experiment hypothesized that AQP4 inhibition reduces inflammation and apoptosis after brain I/R by regulating the ERK1/2 pathway.

**Fig. 1:**
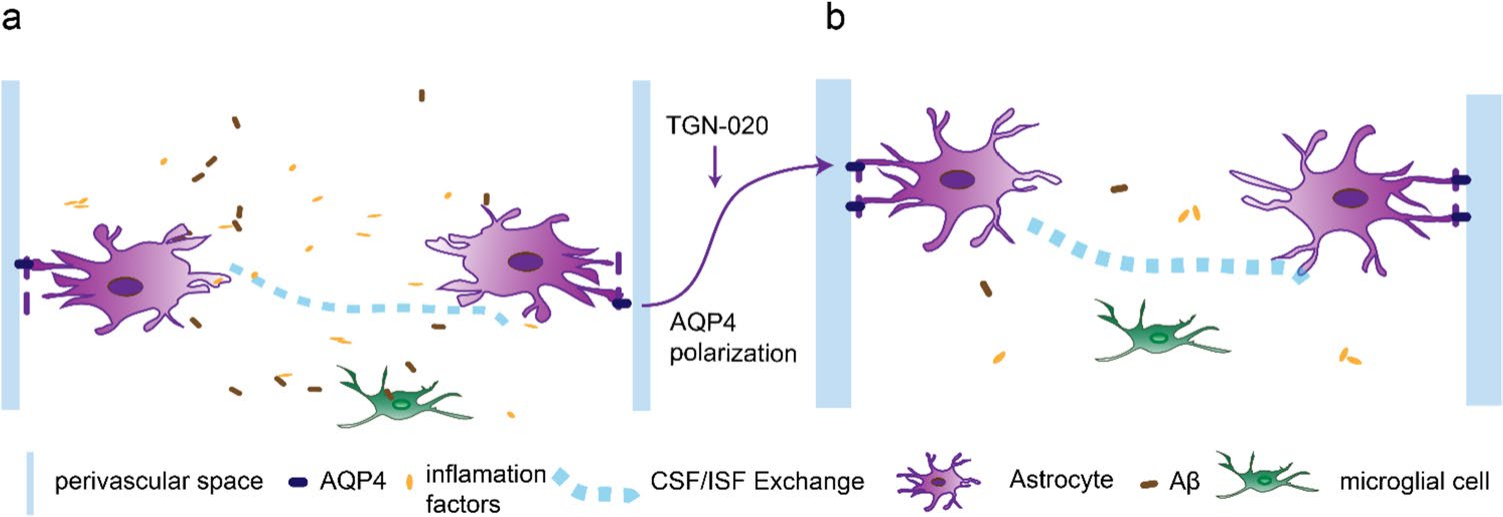
**a** Schematic representation of glymphatic flow following cerebral ischemia. Post-I/R glymphatic dysfunction triggers reactive astrogliosis and augments AQP4 mislocalization. Consequently, it disrupts the flow of CSF-ISF, leading to substantial deposition of Aβ protein within the glymphatic system. Moreover, glymphatic dysfunction post-I/R contributes to an elevated release of inflammatory cytokines from inflammatory cells. **b** Schematic representation of glymphatic flow post-TGN-020 treatment. AQP4 polarization reverts to astrocyte terminals, promoting CSF-ISF flow and leading to a minimal amount of Aβ protein and inflammatory factors deposited within the brain parenchyma

The AQP4-specific inhibitor, TGN-020, has demonstrated the ability to amplify AQP4 polarization and activation of astrocytes subsequent to I/R [22, 23]. TGN-020 has demonstrated a reduction in AQP4 polarization in neurodegenerative disease models, including Alzheimer’s disease and Parkinson’s disease [24, 25], thereby exacerbating glymphatic dysfunctions in which Aβ metabolism plays a crucial role. Intriguingly, multiple studies have revealed that in I/R models, TGN-020 treatment can reinstate AQP4 polarization in brain astrocytes post-I/R. Consequently, a deeper understanding of TGN-020’s impact on AQP4 polarization shifts following cerebral I/R and its implications for glymphatic function is paramount for cerebral I/R interventions. We hypothesize that post-brain I/R, alterations in AQP4 polarization, and glymphatic dysfunction impair CSF flow, leading to compromised secretion of inflammatory cytokines and Aβ. TGN-020 ameliorates post-I/R glymphatic dysfunction, leading to elevated secretion of inflammatory cytokines and Aβ in the CSF (Fig. 1a, b). In addition, after cerebral I/R, the activation of the ERK1/2 pathway occurs. However, the introduction of TGN-020 inhibits this activation. As a result, neurological deficits are reduced, and both inflammatory cells and inflammatory factors are diminished. In this study, we used TGN-020 to inhibit AQP4 expression and identified differentially expressed genes through RNA-seq analysis. We employed GO and KEGG analyses to elucidate the impact of TGN-020 on inflammation and apoptosis post-cerebral I/R and to confirm the related mechanisms.

## Methods

### Animals

This study was approved by the Animal Care and Use Committee of Guilin Medical University. Adult (20–30 g) C57/BL6 male mice (Hunan Slack Co., Ltd.) were used in this experiment. The animals were kept in an automatic 12-h cycle of light and dark, and food and water were freely available, randomly assigned to I/R, I/R + TGN-020 treatment, or sham groups.

### Surgical Procedures and Treatments

The anesthetized mice were placed in the supine position with a dental rest and their limbs bound. The skin was dissected at the midline of the neck, and the muscles and fascia were separated to expose the right internal carotid artery (ICA), external carotid artery, and common carotid artery. The body temperature of the animals was upheld at 37 °C through the use of heating pads. Then, polylysine-coated nylon wire (2541, Leap Biology, Changsha, China) was inserted into the right arteriole to block blood flow to the arteriole to achieve middle cerebral artery occlusion (MCAO), and reperfusion was achieved by pulling out the embolized wire after 90 min of ischemia. The TGN-020 (MedChemExpress, HY-W008574) treatment group was dissolved in 0.5% CMC-Na and then intraperitoneally injected with TGN-020 (200 mg/kg) 5 min after successful occlusion according to the previous research formula [23], while the control group was only exposed to the right internal carotid artery (ICA), external carotid artery, and common carotid artery. The same dose of 0.9% normal saline was given at the same time point of drug injection. The compound (S)-2-(4-fluorophenyl)-1-(toluene-4-sulfonyl) pyrrolidine (Ro 67–7476) functions as an allosteric enhancer for the metabolized glutamate receptor (mGluR1) activated by glutamate. This enhancer possesses the capability to directly activate the p-ERK1/2 signaling pathway [26]. Ro 67–7467 (obtained from MedChemExpress, catalog number HY-100403) was dissolved in 0.9% normal saline and subsequently administered intraperitoneally at a dosage of 10 mg/kg, 1 day following the injection of TGN-020.

#### Neural Score Measurement

After a span of 48 h following reperfusion, the assessment of neurological function in the mice was conducted in accordance with Longa’s five-stage grading system, which categorizes as follows: 0, absence of defects; 1, inability to extend the left paw; 2, longitudinal rotation; 3, falling towards the left; 4, incapability of autonomous walking. For the subsequent investigations, mice that exhibited neurological scores ranging from 1 to 3 were chosen. Meanwhile, those with scores corresponding to 1 or 4 were categorized as having undergone unsuccessful MCAO surgery [27]. Animals that failed to fulfill any of these criteria within a 24-h window after a stroke were deemed to have spontaneously recovered and were excluded from the analysis.

#### Infarct Volume Measurement

Mouse brains were retrieved and subjected to staining using 2,3,5-triphenyltetrazolium chloride (TTC) obtained from Sigma, USA. This staining was carried out to evaluate the infarct volume 48 h following TGN-020 treatment. The brain tissue was sectioned into slices measuring 1 mm in thickness. These sections were treated with a 2% TTC solution at a temperature of 55 °C for a duration of 5 min. Subsequently, the sections were fixed using 4% paraformaldehyde at a temperature of 4 °C until imaging was performed. The images were captured using a digital camera, and the analysis of infarct volume was executed utilizing the ImageJ software. The calculation of the infarct volume percentage involved determining the discrepancy between the volume of the contralateral hemisphere and the volume not affected by infarction within the same hemisphere. This value was then divided by the volume of the contralateral hemisphere [28].

### RNA Sequencing and Analysis

RNA sequencing (RNA-seq) analysis was performed using BGI Wuhan (Wuhan, China). Purified poly(A) RNA was extracted from total RNA. It was then converted into a double-stranded cDNA, which was then sequenced using a standard procedure according to the manufacturer’s protocol.

### Differential Gene Analysis

Bowtie2 (v2.3.4.3) (http://bowtiebio.sourceforge.net/%20Bowtie2%20/index.shtml) assisted in comparing data to the reference gene set. The reference gene set was provided by Dr. Tom’s Multiomics Data Mining System. The RSEM (v1.3.1) gene expression with the quantitative software (https://github.com/deweylab/RSEM) was used. Moreover, pheatmap (v1.0.8) (https://cran.r-project.org/web/packages/pheatmap/index.html) was used to draw gene expression quantity clustering heat maps in different samples. DESeq2 (v1.4.5) (http://www.bioconductor.org/packages/release/bioc/htmL/for) was used to assess differences in genetic testing, conditions for the *Q* value of 0.05 or less, or FDR 0.001 or less.

### KEGG, Network, and GO Enrichment Analysis

Hypergeometric tests Phyper (https://en.wikipedia.org/wiki/Hypergeometric_distribution) to GO (http://www.geneontology.org/) and KEGG genetic variations (https://www.kegg.jp/) were used to further explore gene function related to phenotypic change. For enrichment analysis, *Q* value ≤ 0.05 was taken as the threshold, and those fulfilling this condition were defined as having significant enrichment in candidate genes.

### The Fluorescent Tracer

The glymphatic function was assessed by intracisternal injection of the fluorescent tracer. Mice were anesthetized initially using isoflurane (5% oxygen) and then placed on a stereotactic framework, with the anesthetic maintained at approximately 1.5% (oxygen). The posterior atlanto-occipital membrane was surgically exposed, and a 32-G needle attached to a Hamilton syringe was inserted into the cerebellar cisterna. The apparatus contains 1% glucan, luciferin, and biotin-labeled 3 kDa soluble lysine fixative (D3305, Invitrogen) dissolved in mouse artificial cerebrospinal fluid (Harvard Apparatus) and injected at a concentration of 5 µg/µL in 5 min (the total volume is 10 µL) at a rate of 2 µL/min. The needle was immobilized for 10 min and then removed, and the atlanto-occipital membrane was sealed to avoid any CSF reflux. The brain was sliced into sections measuring 100 µm in thickness. The inflow of the tracer was monitored through employment of a laser scanning confocal microscope (NIS-elements, AX, Tokyo, Japan).

### To Observe the Effect of Solute Drainage in Interstitial Brain

A tracer labeled as 70 kDa Rhodamine (RB)-Dextran (RuixiBiotechCo.Ltd, R-TRD-005) was dissolved in artificial cerebrospinal fluid to attain a concentration of 2%. This solution was then injected into the brain parenchymal lesion with a volume of 2 µL, utilizing a stereotactic apparatus, for a duration of 10 min. The injection coordinates were set as follows: anterior/posterior (AP) 1.5 mm; medial/lateral (ML) 1.5 mm; dorsal/ventral (DV) 2.5 mm from the CSF. To prevent any potential backflow, the syringe remained in place for 5 min following each injection. The mice were administered deep peritoneal anesthesia, succeeded by infusion of 0.9% saline via cardiac means, and subsequently perfused with a solution of 4% paraformaldehyde using a perfusion pump (Cole-Parmer, USA). After dissection, the brain was securely immobilized at a temperature of 4 °C for the duration of one night. Subsequently, the brain was sectioned into slices measuring 100 µm in thickness. These slices were utilized to observe the outflow of the tracer through employment of a laser scanning confocal microscope (NIS-elements, AX, Tokyo, Japan).

### Immunofluorescence (IF)

Mice were infused using phosphate buffers, followed by 4% paraformaldehyde, and their brains were excised and immobilized overnight at 4 °C. After dehydration, wax leaching, embedding, and sectioning, three consecutive coronal brain sections (thickness: 4 µm) were obtained from each animal at approximately 0.24 mm relative to the fontanelle. Glial fibrillary acidic protein (GFAP, 1:500, Proteintech, Beijing, China); anti-Iba-1 (1:500, A19776, ABclonal, Wuhan, China); anti-cellular communication network factor 1 (CCN1) (1:100, Proteintech, Beijing, China); anti-Aβ1–42 (1:100, ab201060, Abcam, USA); and anti-AQP4 (1:300, Proteintech, Beijing, China) antibodies were selected from well-preserved slices and immunostained. Donkey anti-rabbit and anti-mouse antibodies conjugated with Alexa Fluor 488 and 568 (1:200, Proteintech, Beijing, China) were used as secondary antibodies. Finally, the slice with 4′,6-diamidino-2–1, dihydrochloride (DAPI, 1:1000; Sigma-Aldrich, St. Louis, MO, USA) was incubated together. Immunofluorescence sections were scanned using a Vslide scanning microscope (Nikon, Chiyoda, Tokyo, Japan) using a × 20 primary target. All images were obtained using a constant scan setting, and to characterize the expression patterns of AQP4 and GFAP, further semi-quantitative analysis was performed using ImageJ (National Institutes of Health, Bethesda, MD, USA).

To assess AQP4 expression and polarization in the peri-infarct region, the mean fluorescence intensity of AQP4 emission channels was measured, and AQP4 polarization was calculated as the ratio of low-threshold to high-threshold AQP4-positive areas [23]. The percentage of GFAP immunostaining area (GFAP area %) of RIO was used to analyze reactive astrogliosis. All histological data were standardized by contralateral values and calculated twice to minimize measurement error.

### Western Blot (WB) Analysis

Mouse cerebral cortex tissues were extracted and homogenized in a lysis buffer containing a mixture of protease inhibitors. After centrifugation at 12,000 rpm for 10 min, the supernatant was collected. A rapid Gold BCA Protein Assay Kit (Thermo Scientific, Rockford, USA) was used to determine protein concentrations. The same amount of protein was isolated using 10% SDS-PAGE and then transferred to a PVDF membrane (Billerica Millipore, MA, USA). Anti-CCN1 (1:2000, Proteintech, Beijing, China), anti-Aβ1–42 (1:2000, ab201060, Abcam, USA), anti-IL-1β, anti-TNF-a (1:2000, ab201060, Abcam, USA), and anti-AQP4 (1:2000, Proteintech, Beijing, China) were also used, and anti-cleaved caspase-3 (1:1000, Affinity, China) and anti-p-ERK1/2 (1:2000, Ap0472, ABclonal, Wuhan, China) were cultured overnight at 4 °C. Protein bands were detected using the enhanced chemiluminescence detection system (Cell Signaling Technologies, Beverly, MA, USA), and the ImageJ image analysis program (NIH ImageJ, USA) was used to determine the strength of the immune response bands.

### Tunel Staining

DNA strand breaks in apoptotic cells were used to assess the TUNEL test kit (Beyotime, C0003). After the cells were dewaxed and sliced, they were washed using cold PBS. After penetration, 50 µL of the TUNEL reaction mixture was added to each sample and incubated at 37 °C for 60 min. Finally, the slice was hydrolyzed using 4′,6-diamidino-2-phenylindole, dihydrochloride (DAPI, 1:1000; Sigma-Aldrich, St. Louis, MO, USA) and incubated together. The TUNEL-positive cells were observed in scanning immunofluorescence sections using a Vslide scanning microscope (Nikon, Chiyoda, Tokyo, Japan) with × 20 primary targets.

### Statistical Analysis

All data are expressed as mean ± SD. *p* < 0.05 was considered statistically significant. All data were tested for normality. One-way analysis of variance (ANOVA) and post hoc least significant difference (LSD) tests or two-way ANOVA and post hoc least Geisser-Greenhouse correction were used to compare differences between groups. GraphPad Prism 8.0 (GraphPad Software Inc., La Jolla, CA, USA) was used for data analysis.

## Results

### TGN-020 Improves Cerebrospinal Fluid Flow, AQP4 Polarization, and Aβ Deposition Around Cortical Infarction

**Fig. 2:**
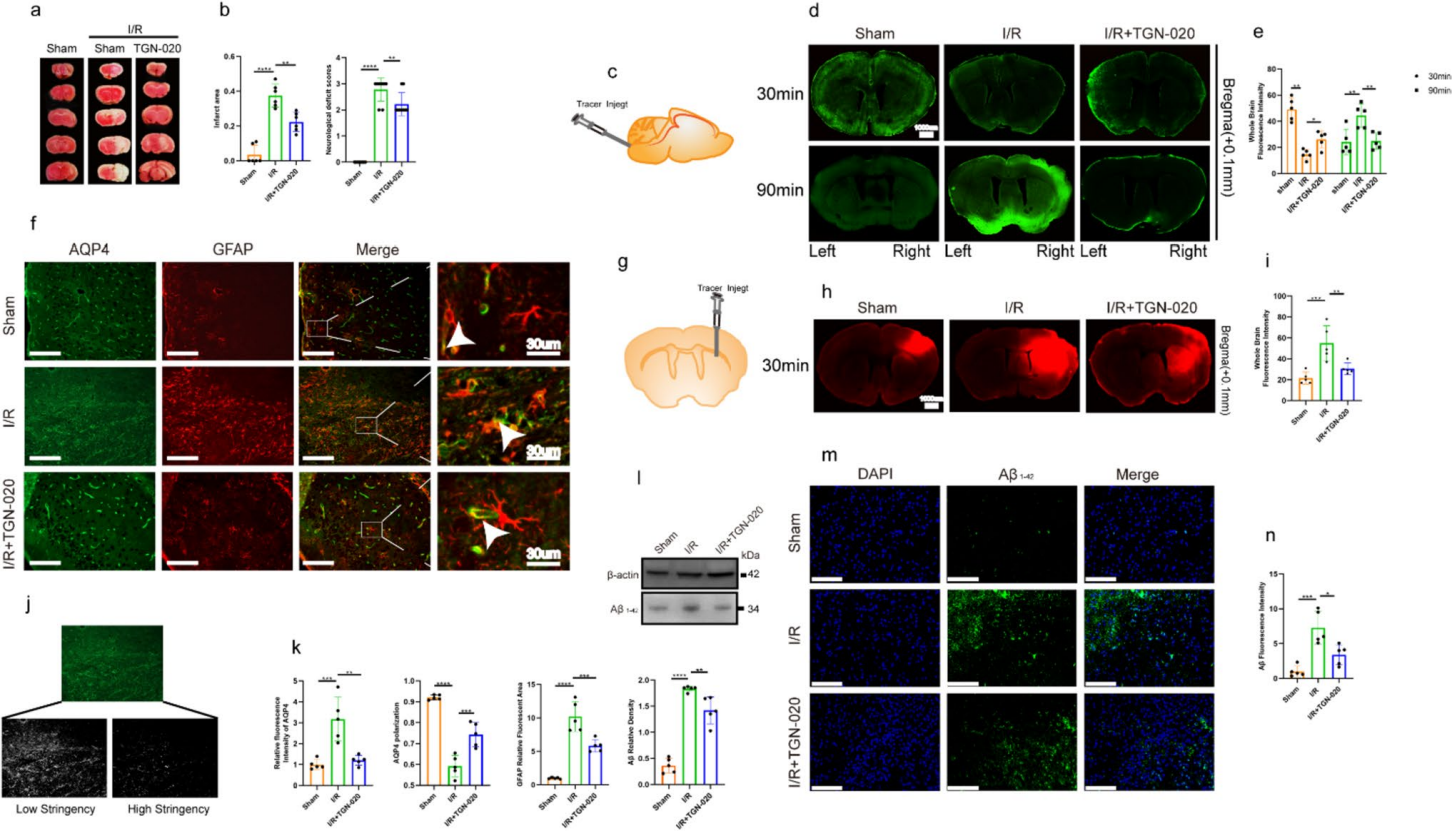
Following a 48-h period I/R, we assessed the infarction area and observed glymphatic alterations surrounding the infarction zone within three distinct groups of mice. **a**, **b** Infarct size (*n* = 6) and neurological function scores (*n* = 9) before and after treatment with TGN-020. **c**, **d** The injection along the cistern of the brain in mice was described. Two-way analysis of variance was used. **e** Representative image of infarct cortical fluorescent tracer inflow (green), *n* = 5. Ratio = 1000 µm. **f** Comparison of GFAP-positive areas (red) and AQP4 polarization (green) in the peri-infarct cortex; the white arrows represent the distribution of AQP4 around the astrocytes, *n* = 5. Ratio = 125 µm. **g**, **i** The visual depiction of interstitial drainage (highlighted in red) was recorded subsequent to introducing the Rodin express tracer into the parenchyma of the infarcted side. *n* = 5. Ratio = 1000 µm. **j** AQP4 high stringency region and low stringency region representation. **k** Statistical map of AQP4 immunofluorescence intensity, AQP4 polarization, GFAP area, and Aβ protein expression. **l**, **n** Immunofluorescence and western blotting showed deposition of Aβ (green) in infarct cortex, *n* = 5. Ratio = 125 µm. One-way analysis of variance was used, and the data were expressed as mean ± SD. *p < 0.05, **p < 0.01, ***p < 0.001

While exploring the potential of TGN-020 treatment to alleviate the effects of cerebral I/R injury, our initial focus was on assessing its therapeutic efficacy in the context of cerebral I/R injury. A notable reduction in infarct volume and an improvement in neurological deficits were observed upon administering TGN-020, as compared to the I/R group (Fig. 2a, b). Following this, we introduced fluorescent tracers 48 h after I/R and proceeded to evaluate the dynamics of CSF flow. This evaluation encompassed the collection of brain tissue samples at two distinct time intervals, specifically 30- and 90-min post-tracer injection (Fig. 2c, d). The outcomes indicated that at the 30-min mark post-tracer injection, the cortex of the control group exhibited a higher presence of tracer in comparison to the I/R group. Notably, the ischemic side of the I/R mouse cortex showed no trace of the tracer, while minimal presence was detected on the contralateral side. This observation hinted at a delay in the influx of the tracer into the CSF. Upon subjecting the samples to TGN-020 treatment, a modest amount of tracer was discernible on the ischemic side, accompanied by an augmented influx on the contralateral side when compared to the I/R group. This indicated an enhancement in CSF inflow (Fig. 2d, e). However, at the 90-min post-injection mark, the tracer within the control group had been nearly entirely cleared, while substantial tracer accumulation was visible on the ischemic side of the I/R group, with slight accumulation also present on the contralateral side. Upon TGN-020 treatment, both sides exhibited decreased tracer accumulation (Fig. 2d, e), suggesting that TGN-020 intervention supports post-I/R CSF clearance. The perivascular distribution of tracer on the infarct side was visualized more clearly under a high-magnification microscope (Figure S1 a). In order to delve deeper into the assessment of CSF clearance, we conducted an injection of the tracer into the brain parenchyma, closely monitoring the subsequent outflow of the tracer within the brain tissue (Fig. 2g). After a circulation period of 30 min and subsequent sample processing, we meticulously analyzed representative sections to ascertain the extent of residual tracer within the brain parenchyma. The findings disclosed a significantly lower residual tracer in the TGN-020 treatment group in contrast to the I/R group (Fig. 2h, i), underscoring a more pronounced trend of tracer drainage in the treated group as opposed to the untreated one.

Further examination involved the impact of TGN-020 on the polarization of AQP4 subsequent to brain I/R. This was pursued through the assessment of AQP4 and GFAP expressions in the peri-infarction cortex 48 h after brain I/R. Results highlighted an increase in the proliferation of GFAP-labeled astrocytes following I/R. However, relative to the reperfusion group, both the number and volume of astrocytes were reduced in the TGN-020 treated group (Fig. 2f), suggesting that TGN-020 inhibited astrocyte proliferation post-I/R. In the I/R group, AQP4 was primarily localized in the nerve fibers, losing the typical positioning of astrocyte foot processes, while in the TGN-020 treatment group, AQP4 was predominantly localized in the perivascular area, closely resembling the polarized distribution in the control group (Fig. 2f). Statistical analysis demonstrated a decrease in AQP4 polarization following I/R, and TGN-020 treatment enhanced cortical AQP4 polarization relative to the I/R group (Fig. 2k). To further explore the impairment of glymphatic waste excretion caused by obstructed CSF influx, brain tissue was harvested 48 h post-I/R, and the cortical Aβ content was measured using Western blotting and immunofluorescence (Fig. 2l, m). As depicted, cortical infarct-related Aβ accumulation was significantly decreased following TGN-020 treatment, and cortical homogenate analysis revealed a reduction in Aβ content post-TGN-020 treatment. Therefore, our experiments suggest that TGN-020 can mitigate glymphatic dysfunction following cerebral I/R.

### Differential Expression Genes (DEGs) of the I/R and TGN-020 Groups Screened

**Fig. 3:**
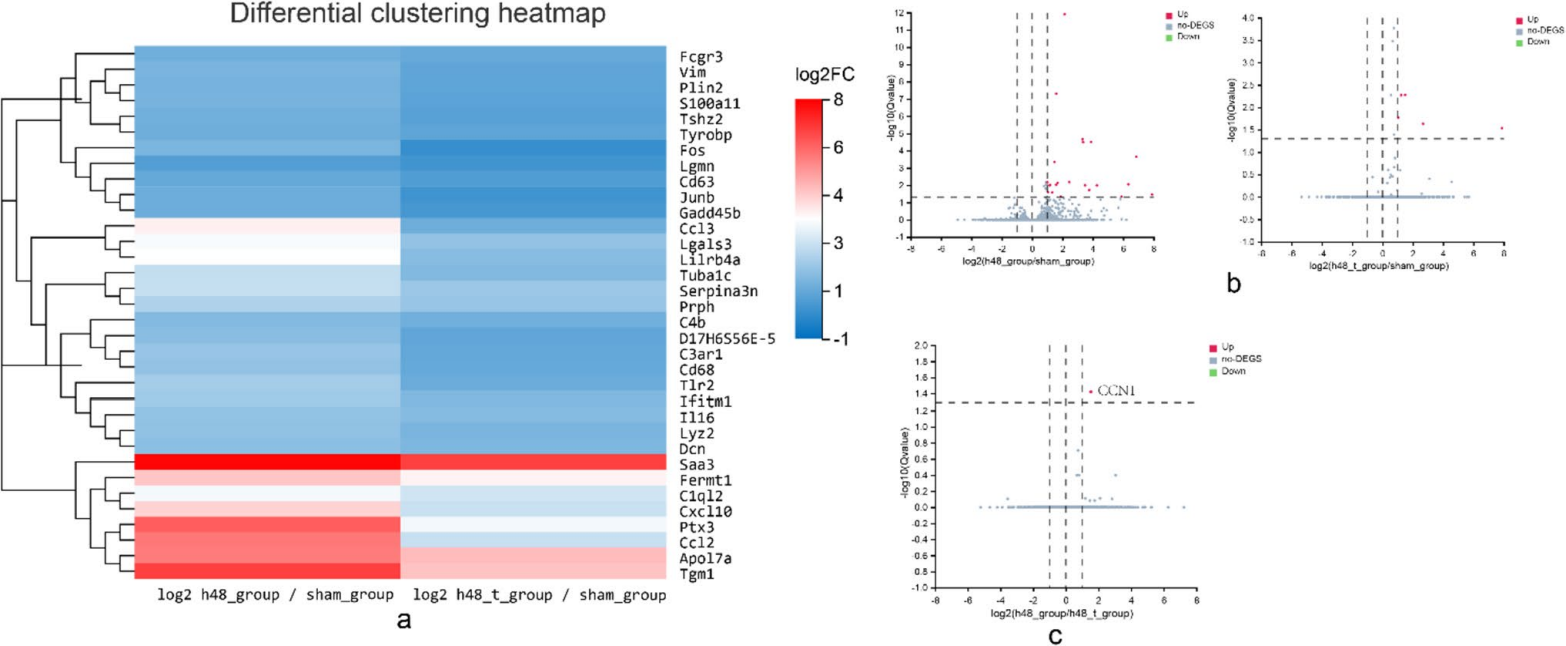
TGN-020 treatment of DEGs, *n* = 3. **a** Heat map of RNA sequence results. **b**, **c** The volcano maps of candidate DEGs in microarray datasets based on screening criteria. The expression of CCN1 is down-regulated and marked in red

Previous studies confirmed that TGN-020 enhances lymphoid function, setting the foundation for further exploration of its neuroprotective mechanism on cerebral I/R mice. Gene expression levels in the peri-infarct cortex, examined 48 h post-I/R and TGN-020 treatment, are illustrated in the heat map (Fig. 5). We identified 34 down-regulated genes, primarily linked to inflammatory apoptosis, including GFAP, interleukin 16 (IL-16), C–C motif chemokine ligand 3 (Ccl3), and C–C motif chemokine ligand 2 (Ccl2) (Fig. 3a). According to *q* < 0.05 and | log (multiple) |> 1, further volcano plots were used to identify up-regulated and down-regulated genes. Among them, 29 genes were up-regulated after I/R and 9 genes were up-regulated after TGN-020 (Fig. 3b). Notably, CCN1 expression significantly decreased in both the I/R group and I/R + TGN-020 group (Fig. 3c).

### Functional Annotation and Pathway Enrichment Analysis of DEGs

**Fig. 4:**
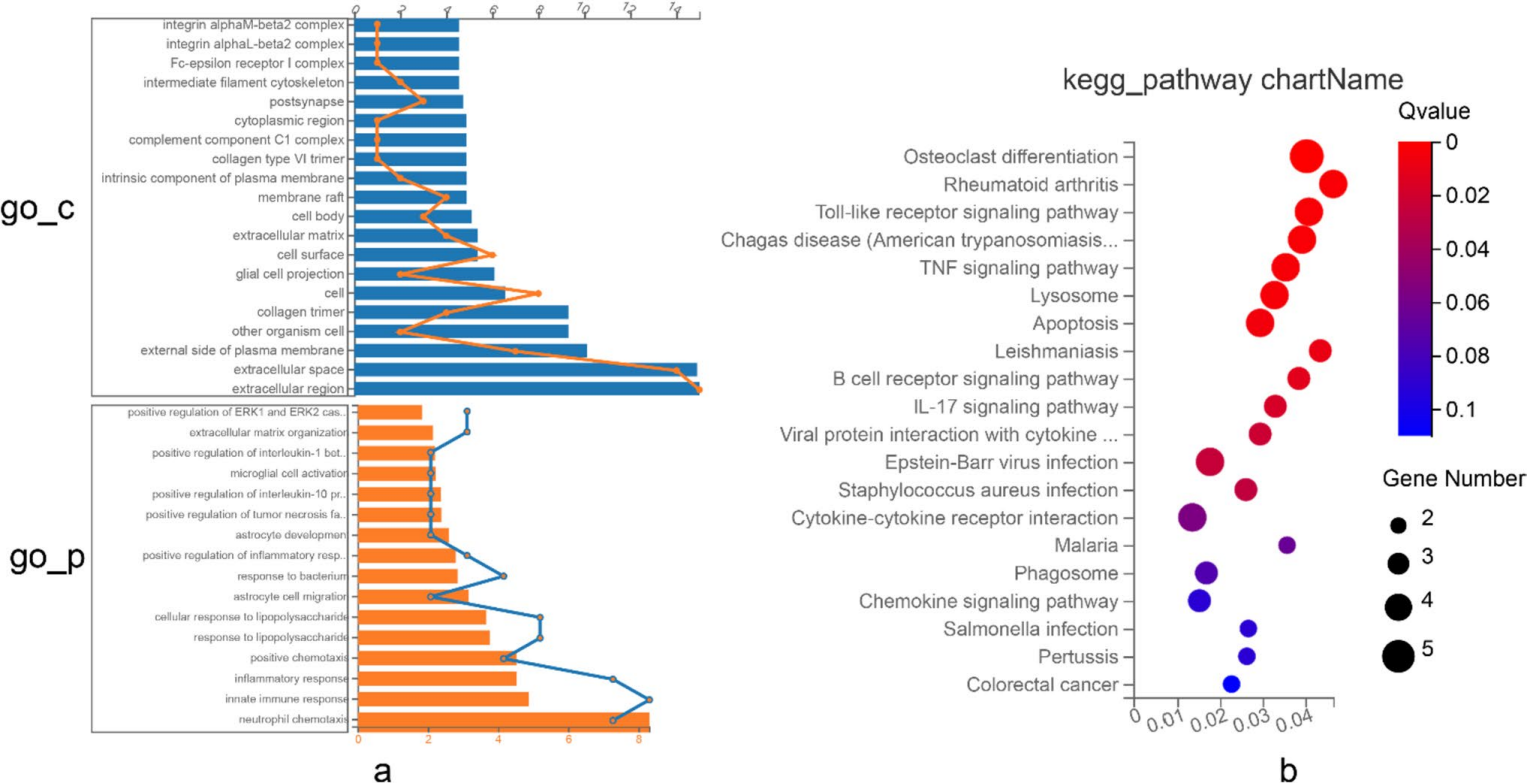
Analysis of DEG functional annotation and pathway enrichment, *n* = 3. **a** GO functional annotation revealed that DEGs were enriched in three aspects: BP in yellow and cell component (CC) in blue. **b** KEGG pathway enrichment results

After identifying DEGs in the treatment group, we employed GO analysis for functional annotation and KEGG analysis for pathway enrichment to investigate the underlying biological mechanism (Fig. 4). The GO analysis primarily revealed enrichment for biological processes (BP) and cellular components (CC). Primarily, DEGs are involved in regulating the extracellular matrix and Aβ deposition within the cellular components (CC). KEGG analysis indicated that the DEGs are primarily associated with apoptotic and inflammatory pathways.

### TGN-020 Alleviates Brain I/R Inflammation and Apoptosis Through Glymphatic and ERK1/2 Signaling Pathways

**Fig. 5:**
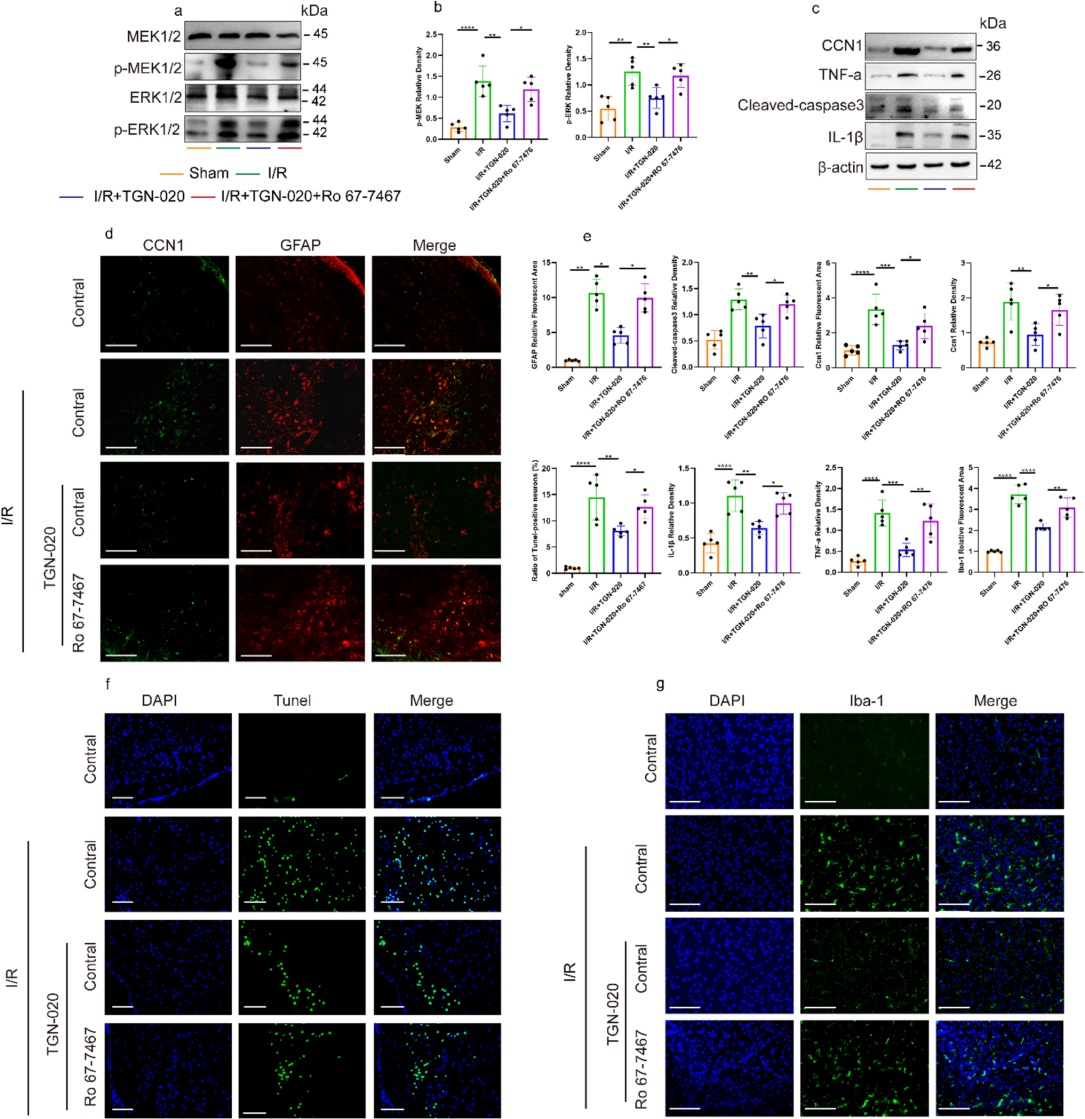
The expression patterns of inflammation and apoptosis in microglia and astrocytes in the cortex surrounding infarction were studied. **a**, **b** Western blot showed the protein bands of p-MEK, p-ERK, MEK, and ERK, *n* = 5, and the differences were statistically significant. **c**, **e** WB was used to detect the expression levels of CCN1, TNF-a, IL-1β, and cleaved caspase-3 protein in the infarct-side cortex. Fluorescence areas of Iba-1, GFAP, Ccn1, and TUNEL-positive cell rate were statistically significant, *n* = 5. **d** GFAP (red) and CCN1 (green) immunostaining of peri-infarct cortex in mice, *n* = 5. Ratio = 125 µm. **f** Comparison of TUNEL staining in the peri-infarct cortex of the four groups, *n* = 5. Ratio = 50 µm. **g** DAPI (blue) and Iba-1 (green) immunostaining of infarct cortex in TGN-020 and control groups, *n* = 5. Ratio = 125 µm. One-way analysis of variance and LSD post hoc test were used for statistical methods, and all data were expressed as mean ± SD. *p < 0.05, **p < 0.01, ***p < 0.001

Our findings demonstrated that TGN-020 significantly reduced the expression of p-ERK1/2 and p-MEK1/2, while the expression of p-ERK1/2 and p-MEK1/2 increased post-activator use (Fig. 5a). TGN-020 reduced microglial and astrocyte activation in the peri-infarction cortex post-I/R, contrary to the ERK1/2 pathway activation which increased their activation (Fig. 5d, h). The expression levels of inflammatory cytokines TNF-α, IL-1β, and CCN1 in the peri-infarction cortex showed an increase post-I/R, a decrease post-TGN-020 treatment, and a subsequent increase following ERK1/2 pathway activation (Fig. 5c). Additionally, TGN-020 treatment led to a decrease in the expression of apoptotic cells and cleaved caspase-3 promoter (Fig. 5c, f). Expression of apoptotic cells and cleaved caspase-3 saw an increase following the application of ERK1/2 pathway activators. The outcomes of this study suggest that TGN-020 has the capacity to alleviate inflammation in microglial cells and astrocytes, along with reducing brain apoptosis subsequent to I/R. These effects are believed to be mediated by the ERK1/2 signaling pathway. Studies indicate astrocytes as the source of CCN1 secretion, given that CCN1 is less thoroughly examined in brain tissue. Immunostaining for GFAP and CCN1 revealed that CCN1 was predominantly localized around astrocytes (Fig. 5d), with a consistent expression trend, reinforcing our study. Therefore, we propose that TGN-020 could play a role in mitigating inflammation and apoptosis following cerebral I/R injury via the glymphatic system.

## Discussion

Consistent with prior studies, acute AQP4 inhibition using TGN-020 decreased infarct volume 2 days post-stroke and reduced peri-infarct AQP4 polarization [23]. Moreover, our study illustrates that glymphatic dysfunction is closely linked to the activation of astrocytes and microglia. Enhanced AQP4 polarization post-I/R led to reduce expression of IL-1β, TNF-a, and cleaved caspase-3 through ERK1/2 pathway inhibition.

Of significance, a particular study revealed that the introduction of contrast agents into the cisterna yielded a notable delay in ventricular inflow, even on the uninfarcted contralateral side [29]. Similarly, within the context of our experiments, we discerned disruptions in the contralateral CSF inflow. This observation pointed towards a disturbance in bilateral CSF inflow subsequent to cerebral I/R. This phenomenon might stem from the interplay between blood flow dynamics and metabolic processes spanning the two hemispheres of the brain. This interaction implies that unilateral cerebral I/R could potentially exert an influence on the function of the medullary glymphatic system on the contralateral side.

The accumulation of Aβ peptide in the brain parenchyma is one of the main contributors to neuroinflammation and cognitive decline [30]. Recent findings suggest a significant role of the glymphatic system in Aβ clearance [31]. Our research proved that TGN-020 accelerates Aβ clearance and reduces Aβ peptide accumulation in the brain parenchyma by enhancing ISF drainage after I/R, thereby contributing to the alleviation of neuroinflammation induced by Aβ deposition. As mentioned earlier, AQP4 polarization at the perivascular astrocyte endfeet exerts the most significant influence on glymphatic function. In the context of ischemic stroke, peri-infarct reactive astrocyte hyperplasia is commonly associated with AQP4 depolarization in the same region [32–34]. Our investigation reveals that TGN-020 therapy reinstates AQP4 polarity subsequent to cerebral I/R injury. This finding aligns with another study that explored I/R injury, wherein TGN-020 was also observed to reinstate AQP4 polarity [23]. This latter study suggested that the reduction in astrocyte proliferation resulting from acute AQP4 inhibition could contribute to the preservation of AQP4 polarity. However, in cases of Parkinson’s disease, TGN-020 treatment stimulates astrocyte proliferation [25], which may lead to further reduction of AQP4 polarization. Prior research has noted heightened AQP4 expression post-cerebral infarction, and AQP4 inhibition has been demonstrated to exert protective effects in models of cerebral infarction [22]. In scenarios involving subarachnoid hemorrhage, AQP4 expression tends to decrease. The augmentation of AQP4 expression might facilitate AQP4 polarity, thereby playing a beneficial protective role within the brain [12]. These observations elucidate the reason behind TGN-020’s capability to restore AQP4 polarity, despite inhibition of expression in the context of cerebral infarction. Additionally, the positioning of AQP4 within the paravascular region serves as a pivotal indicator of AQP4 polarity [12]. Studies have validated that acute AQP4 inhibition via TGN-020 after cerebral I/R enhances paravascular drainage and angiogenesis [35], thereby potentially facilitating the restoration of AQP4 polarity and the elimination of brain metabolic waste, including Aβ proteins. However, there exists a contradictory report indicating that a single high-dose administration of TGN-020 reduces perivascular Aβ40 clearance when compared to normal mice [36]. This finding contradicts our own. Nevertheless, within the context of a brain I/R model, we propose that TGN-020 might enhance paravascular drainage and encourage Aβ clearance by reinstating post-I/R astrocyte hyperplasia and AQP4 polarity.

Recently, an increasing number of studies have highlighted the reciprocal regulation between the glymphatic system and inflammation. Disruptions in the glymphatic system can impact the activation of microglial cells and hinder the clearance of TNF-α, IL-1β, and other related factors in the brain [37]. Conversely, excessive ROS accumulation triggers the activation of inflammasomes in microglia, leading to the release of inflammatory cytokines such as TNF-α, interleukin-1a, and prostanoid prostaglandin E2. These cytokines can impact the clearance capabilities of the glymphatic system [37–39]. Additionally, microglial activation induces NOD-like receptor thermal protein domain-associated protein 3 (NLRP3) inflammasome overexpression and triggers the release of various cytokines. The activity of the NLRP3 inflammasome might impact the glymphatic system by modulating the release of extracellular adenosine triphosphate from astrocytes [40, 41]. Previous research has revealed evidence of astrocyte-microglia crosstalk that can instigate neuroinflammation [42]. In this experiment, we observed microglial and astrocyte activation post-I/R, implying a possible “coactivation” between these activated cells. This coactivation appears to be significantly reduced by the enhancement of AQP4 polarization. These findings suggest that glymphatic dysfunction might impact the crosstalk between microglia and astrocytes via specific mediators.

To illustrate the close relationship between glymphatic enhancement and the regulation of inflammation in astrocytes and microglia, we employed TGN-020 to boost glymphatic function. We found that the down-regulated genes primarily related to infection and inflammation pathways, indicating that glymphatic enhancement may contribute to the down-regulation of inflammation-associated genes. The down-regulated DEGs, including GFAP, CCN1, IL-16, Ccl3, and Ccl2, were predominantly associated with inflammation and the proliferation of astrocytes and microglia, thereby exemplifying the positive influence of glymphatic function on post-I/R inflammation.

CCN1, in conjunction with other extensively conserved homologs, forms the CCN family, encompassing six distinct mammalian members. Early investigations concerning CCN1 have showcased its intimate connection with the extracellular matrix following secretion. Moreover, it has been shown to promote cell adhesion through direct binding to integrin receptors [43]. Recent studies reveal that CCN1, a dynamically expressed multifunctional stromal cell protein, plays a crucial role in cardiovascular system maturation during embryonic development, and regulates inflammation, wound healing, and fibrosis in adults [43, 44]. CCN1 serves as an early biomarker for myocardial infarction, kidney injury, and liver I/R injury [45–47]. CCN1 can exacerbate astrocyte activation, inflammation, and apoptosis following hepatic I/R injury [47, 48]. Brain infections with Zika virus can influence the secretion of CCN1 by astrocytes. However, for the first time, our study demonstrated that CCN1 expression increased after I/R, and TGN-020 inhibited the secretion of CCN1 by astrocytes. This implies a potential mechanism through which CCN1 regulates inflammation and apoptosis following I/R.

This study does have some limitations. Firstly, the AQP4 inhibitor TGN-020 is not glymphatic-specific, and it can simultaneously alter AQP4 expression and polarization. Thus, it remains uncertain whether TGN-020 regulates inflammation and apoptosis via the glymphatic system; future experiments aim to address this limitation. Secondly, it is important to note that all the experiments were conducted solely at a single time point in the initial stage. This limitation prevents us from observing dynamic alterations or the prolonged impacts of TGN-020 on the modulation of inflammation and apoptosis. In addition, although early use of drugs after brain I/R promotes Aβ excretion, changes in cognitive dysfunction cannot be evaluated in the short term, so it is not possible to determine whether TGN-020 improves cognitive impairment after I/R. Future research will be undertaken to delve deeper into the anatomical foundation of glymphatic and meningeal glymphatic vessels within the nervous system.

### Supplementary Information

Below is the link to the electronic supplementary material.

Supplementary file1 (DOCX 599 KB)

## Data Availability

The datasets generated and analyzed during the current study are not publicly available, but are available from the appropriate authors upon reasonable request.
