# Supplementary material for: TGN-020 Alleviate Inflammation and Apoptosis After Cerebral Ischemia–Reperfusion Injury in Mice Through Glymphatic and ERK1/2 Signaling Pathway
Source: Mol Neurobiol. 2023 Sep 11;61(2):1175–86. doi: 10.1007/s12035-023-03636-w (PMC10861636; doi:10.1007/s12035-023-03636-w)
Supplement: Supplementary file 1 — Supplementary file1 (DOCX 599 KB) [file 12035_2023_3636_MOESM1_ESM.docx]

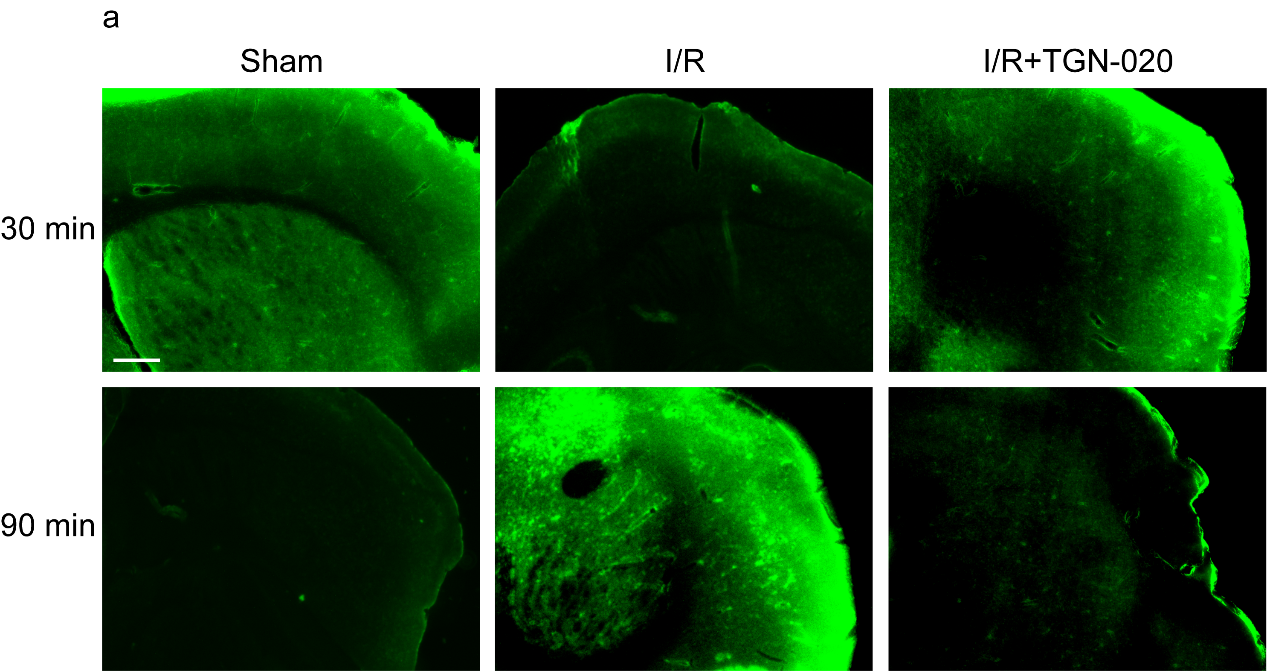


Fig. S1. High magnification images showcasing the distribution of tracer around the cerebrovascular division following TGN-020 treatment post-infarction. a. Representative images illustrating the cortical tracer distribution after 30 and 90 minutes of tracer circulation within the brain. Scale bar = 200µm. n=5.
